# Human papillomavirus-related esophageal cancer survival: A systematic review and meta-analysis

**DOI:** 10.1097/MD.0000000000005318

**Published:** 2016-11-18

**Authors:** Lanwei Guo, Shuzheng Liu, Shaokai Zhang, Qiong Chen, Meng Zhang, Peiliang Quan, Xi-Bin Sun

**Affiliations:** Department of Cancer Epidemiology, Henan Office for Cancer Control and Research, The Affiliated Cancer Hospital of Zhengzhou University, Henan Cancer Hospital, Zhengzhou, China.

**Keywords:** esophageal cancer, HPV, meta-analysis, prognosis

## Abstract

**Background:**

Human papillomavirus (HPV) has been identified to be related to progression of esophageal cancer. However, the results remain controversial. A meta-analysis of epidemiologic studies was therefore conducted to address this issue.

**Methods:**

The electronic databases of MEDLINE and Excerpta Medica database were searched till April 30, 2016. Study-specific risk estimates were pooled using a random-effects model.

**Results:**

Ten studies involving a total of 1184 esophageal cancer cases were included in this meta-analysis. The pooled hazard ratio comparing HPV-positive to HPV-negative esophageal cancers was 1.03 (95% confidence interval 0.78–1.37), which was not significantly correlated with improved survival. However, HPV-16-positive patients might have a significantly favorable survival (hazard ratio 0.73, 95% confidence interval 0.44–1.21).

**Conclusion:**

The meta-analysis indicated that HPV infection may not be of prognostic utility in the evaluation of factors contributing to esophageal cancer. Further large prospective studies are encouraged to stratify survival analysis by HPV type.

## Abstract 2

Supplemental Digital Content is available in the text

## 1 Introduction

Esophageal cancer is the eighth most common cancer globally, with an estimated 455,784 new cases in 2012, and the sixth most common cause of death from cancer, with an estimated 400,156 deaths.^[1]^ Furthermore, the number of esophageal cancer deaths will have increased to 728,945 by the year 2035.^[1]^

The etiology of esophageal cancer remains unclear. Risk factors such as smoking and alcoholism,^[2]^ lack of nutrition,^[3]^ and some chemical factors,^[4]^ and also physical factors (the ingestion of coarse or hot food)^[5]^ were found by epidemiological studies. Infectious agents have also been suggested as direct carcinogens or promoters in esophageal carcinogenesis. Infection with high-risk human papillomavirus (HPV) has been identified as a causal agent in cancers of some site, including cervix, anogenital region, head, and neck.^[6–8]^ As reported previously, the high-risk HPV prevalence was 89.7% in cervical cancer,^[9]^ 29.5% in head and neck cancer,^[10]^ and 22.2% in esophageal cancer.^[11]^

The infection status of HPV may be associated with the prognosis of esophageal cancer based on current studies.^[12–16]^ One study reported that cervical cancer patients who were infected with HPV had a significantly better survival than those who were not while they were receiving radiation therapy.^[12]^ Some retrospective clinical studies have consistently proved that patients with HPV-positive head and neck squamous cell carcinoma (HNSCC) had a better prognosis than patients with HPV-negative tumors.^[13–16]^ Esophagus can be infected with HPV in the same way as the oral cavity, tonsils, and pharynx; it is assumed that the histological similarities between the head and neck squamous epithelia and esophagus would suggest a similar association and clinical characteristics. The prognostic value of the HPV status has previously been investigated in patients with esophageal cancer. However, the results are much controversial.

Therefore, this systematic review and meta-analysis is conducted to clarify the association between HPV infection and overall survival (OS) in esophageal cancer patients.

## 2 Materials and methods

The methods were carried out in accordance with the approved guidelines. This study was approved by the ethics committee of Henan Cancer Hospital.

### 2.1 Literature search strategy

A systematic search was conducted in Excerpta Medica database (EMBASE) and MEDLINE to identify relevant papers. Time range was from the founding of each database to April 30, 2016. Combinations of search terms for HPV or HPV, esophageal neoplasms, and prognosis or prognostic or survival were used (Supplementary 1). Additional relevant references cited in review articles were also assessed.

### 2.2 Inclusion and exclusion criteria

Two authors (SZ and QC) reviewed all papers independently. Any disagreement was resolved by consensus after discussion with a third author (PQ). Inclusion criteria included the following: patients were pathologically diagnosed as esophageal cancer; esophageal cancer OS as the outcome of interest; reported or calculated hazard ratio (HR) values and 95% confidence interval (CI) (or sufficient data to calculate these effect measures); and English articles. For studies which were reported more than once, we used the one which provided more information or published earlier.

### 2.3 Quality assessment

According to a critical review checklist of the Dutch Cochrane Centre proposed by the meta-analysis of observational studies in epidemiology (MOOSE) group, we strictly assessed the quality of all the included studies^[17]^: clear definition of study population and origin of country; clear definition of study design; clear definition of outcome assessment; clear definition of HPV detection method; and sufficient period of follow-up. Otherwise, we would exclude the studies to ensure the quality of the meta-analysis.

**Figure 1:**
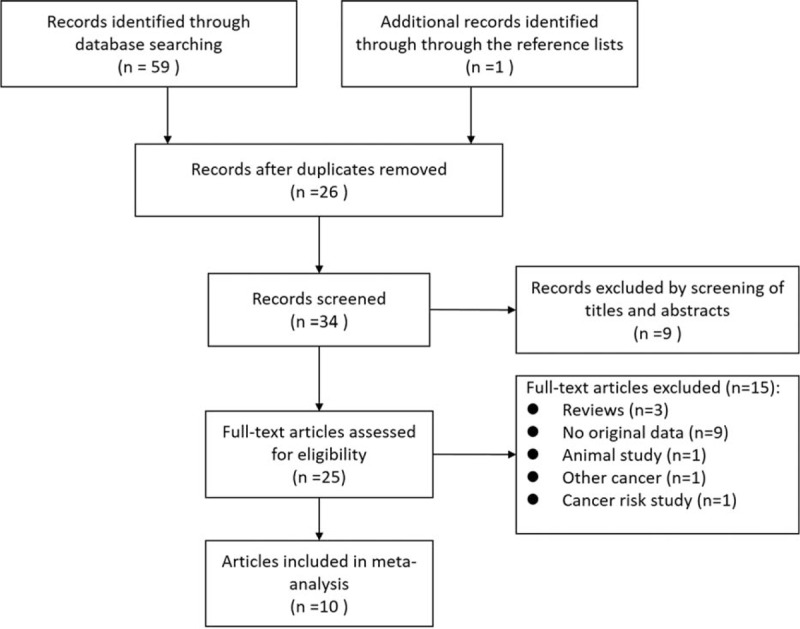
Flow diagram of systematic literature search.

A flow diagram of the study selection process is shown in Fig. 1.

### 2.4 Data extraction

Two of the authors (SL and SZ) extracted data with a standardized extraction form, and any disagreement was resolved by consensus. The following information was extracted from each article: publication year, name of first author, country of origin, study type, period of enrollment, follow-up time, characteristics of the studied population (sample size, age, stage of disease, and treatment method), HPV detection methods, and HR estimates for OS with corresponding 95% CIs. Multivariate Cox proportional-hazards regression analysis was used in the present analysis. When data for HR were not available, we extracted the total numbers of observed deaths and the numbers of patients in each group to calculate HR.^[18]^ Data were extracted by Engauge Digitizer version 4.1 (http://digitizer.sourceforge.net/) from the graphical survival plots when data were only available as Kaplan–Meier curves,^[19]^ then the estimation of the HR was performed by the described method.^[18]^

### 2.5 Statistical analysis

Meta-analysis calculations were performed using STATA 12.0 for Windows (StataCorp LP, College Station, TX). The HR with 95% CI was used to compute the pooled HPV infections and the OS in esophageal cancer patients. We use a fix-effect or random-effect model to pool the data, based on the Mantel–Haenszel method,^[20]^ and the DerSimonian and Laird method,^[21]^ respectively. Both models offer similar results when between-studies heterogeneity is absent; otherwise the random-effect model is more appropriate. So, we will choose the fix-effect model when between-studies heterogeneity is absent and the random-effect model when between-studies heterogeneity exists.

We used the Cochrane *Q* test and *I*^2^ test to quantify inconsistency.^[22]^ Subgroup analyses for HPV infections and the OS in esophageal cancer patients were subsequently carried out according to the study type, geographical region, number of patients, pathological type, detection method, HPV type, max follow-up time, case diagnosis method, and the source of HR. We also conducted the sensitivity analysis to assess the influence of each study on the pooled HR estimates. Finally, to evaluate any existing publication bias, the funnel plot symmetry was tested, and the Begg adjusted rank correlation test, combined with Egger regression, was applied.

## 3 Results

### 3.1 Literature Search

Our initial literature search yielded a total of 60 citations (Fig. 1). After screening of titles and abstracts, 25 were considered of potential value and the full text was retrieved for detailed evaluation. Fifteen of these 25 articles were subsequently excluded from the meta-analysis for the following reasons: 3 were reviews, 1 was animal study, 9 did not provide HRs or CIs, 1 was not esophageal cancer, and 1 was cancer risk study. So, 10 studies were eligible and included in this systematic review and meta-analysis.^[23–32]^

### 3.2 Characteristics of the selected studies

Individual characteristics of the included 10 studies are summarized in Table 1. They were published from 1993 to 2015, and involved a total of 1184 esophageal cancer cases. Among these studies, 6 studies were conducted in China,^[23,26,28–30,32]^ 1 in Japan,^[31]^ 1 in Sweden,^[24]^ 1 in Australia,^[25]^ and 1 in Brazil.^[27]^ Of all the selected studies, 3 presented HRs,^[25,26,28]^ whereas in the other 7 studies,^[23,24,27,29–32]^ HRs were absent, and we needed to calculate the *HR*s from the survival curves. One study^[25]^ did not give accurate data for follow-up. The median follow-up period of all studies ranged from 0.17 to 122.50 months.

**Table 1:**
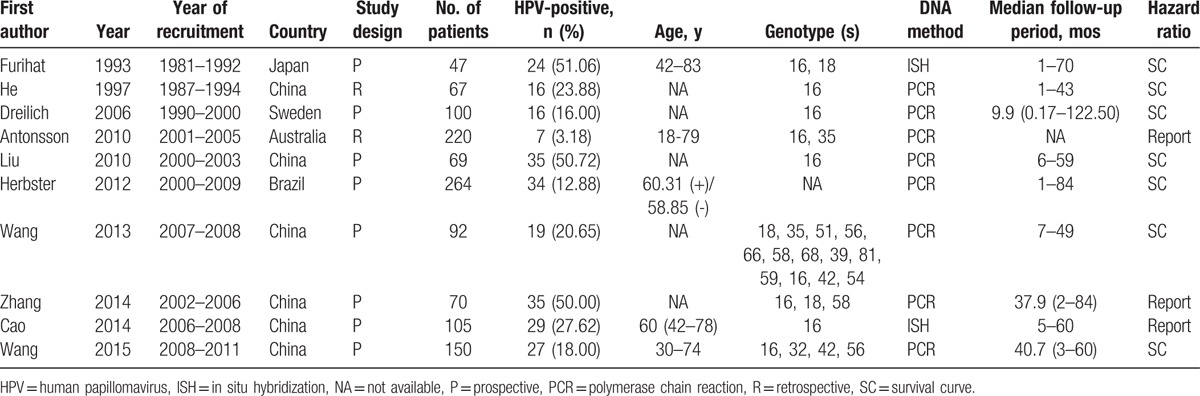
Characteristics of the included studies.

### 3.3 Results of the meta-analysis

**Figure 2:**
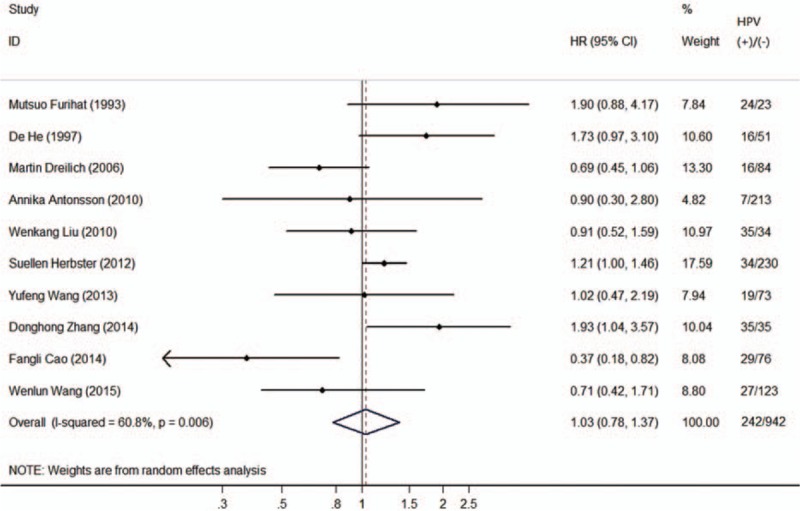
Forest plot comparing HPV-positive to HPV-negative esophageal cancer patients and overall survival. HPV = human papillomavirus.

Among the studies included, five^[24,25,28,29,32]^ showed a negative association comparing HPV-positive to HPV-negative cancers, one^[28]^ of which showed statistical significance; and the other five^[23,26,27,30,31]^ showed positive associations, two^[26,27]^ of which showed statistical significance. The heterogeneity test indicated there was moderate degree of heterogeneity among included studies (*Q* test *P*_heterogeneity_ = 0.006, *I*^2^ = 60.8%), thus a random-effects model was employed to obtain the pooled HR. The pooled HR from the 10 individual effect estimates comparing HPV-positive to HPV-negative esophageal cancers was 1.03 (95% CI 0.78–1.37), which was not significantly correlated with OS (Fig. 2).

#### 3.3.1 Subgroup analyses

Table 2 presents detailed results of subgroup analyses.

**Table 2:**
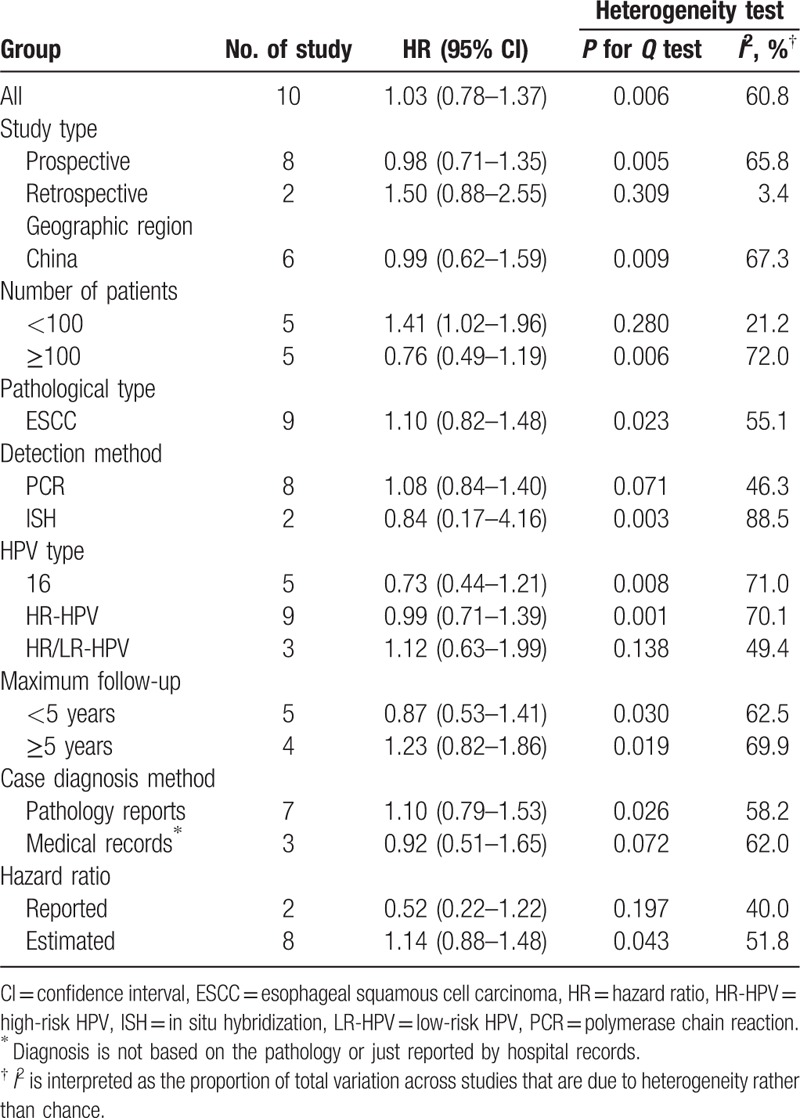
Results of subgroup analyses.

The associations of HPV status and OS in esophageal cancer patients did not differ by study type, geographical region, pathological type, detection method, HPV type, maximum follow-up time, case diagnosis method, and the source of HR. HPV status was significantly associated with poor OS in esophageal cancer patients for studies less than 100 (HR 1.41, 95% CI 1.02–1.96), but did not show statistically significant negative association for more samples (HR 0.76, 95% CI 0.49–1.19). When cancer cases were stratified by HPV type, the pooled HR comparing HPV-16-positive to HPV-16-negative cancers was 0.73 (95% CI 0.44–1.21), which was not significantly correlated with OS. In short, the estimated heterogeneity for the included studies decreased to some degree, but did not obliterate.

### 3.4 Influence analysis of individual studies

**Figure 3:**
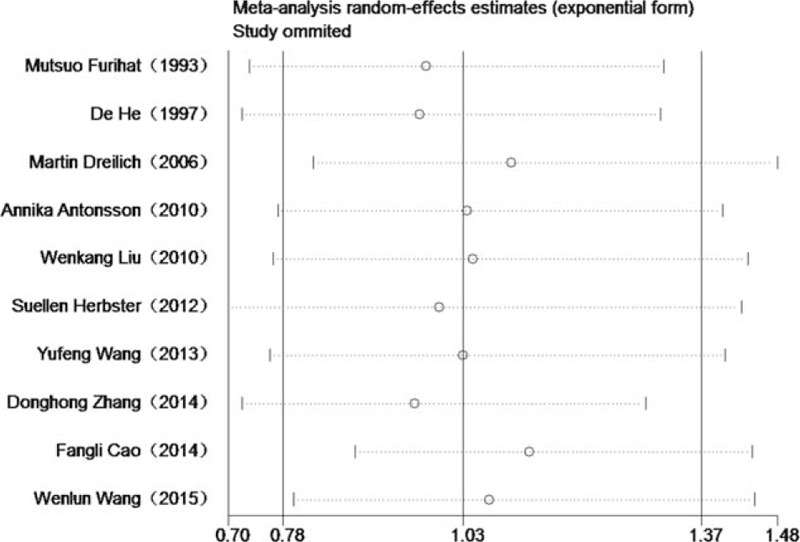
Influence analyses for omitting individual study on the summary HR for overall survival. HR = hazard ratio.

Sensitivity analysis for OS is shown in Fig. 3. The pooled HRs comparing HPV-positive to HPV-negative cancers ranged from 0.97 (95% CI 0.72–1.30) to 1.13 (95% CI 0.88–1.45). The meta-analysis result of the pooled HRs comparing HPV-positive to HPV-negative cancers were not significantly affected by omission of any study included, which indicated that each single study did not influence the stability of pooled HR estimate.

### 3.5 Publication bias

**Figure 4:**
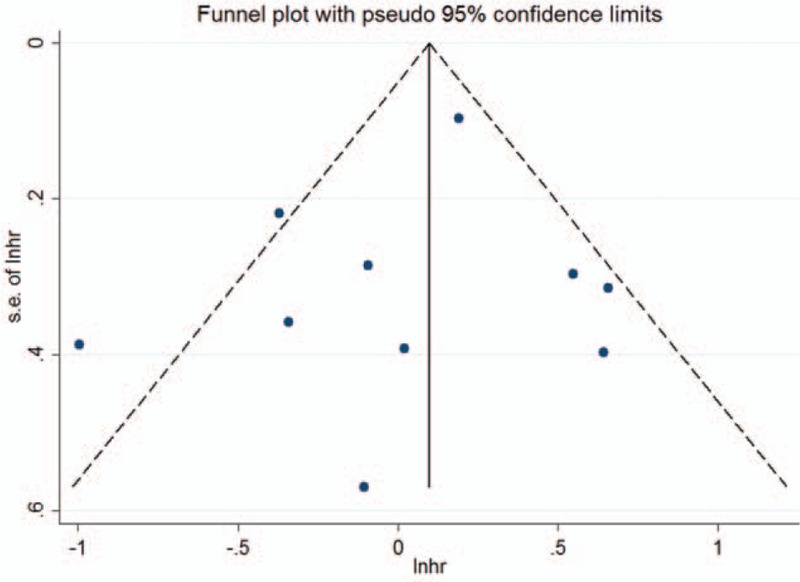
Funnel plots for publication bias of overall survival.

There was no evidence of publication bias as demonstrated by the nonsignificant *P* values of Begg test (0.929), Eegg test (0.528), and the near-symmetric funnel plot (Fig. 4).

## 4 Discussion

As we know, this is the first meta-analysis investigating OS in HPV-related esophageal cancers. Results from this meta-analysis showed that HPV infection was not significantly associated with improved survival in esophageal cancer patients, suggesting that HPV infection may not be of prognostic utility in the evaluation of factors contributing to esophageal cancer.

The association between HPV infection and the occurrence of esophageal cancer was first reported by Syrjanen and Pyrhonen^[33]^ in 1982. Later, many groups studied the relationship between HPV infection and esophageal cancer prognosis. However, the conclusions drawn were inconsistent. It was reported that, in comparison with HPV-unrelated HNSCCs, HPV-positive HNSCCs were associated with a 54% reduction in overall mortality.^[34]^ Unlike HNSCC, the association between HPV status and improved survival in esophageal cancer patients did not exist in this meta-analysis. Among the included studies, only Cao et al^[28]^ demonstrated that patients with HPV-positive esophageal squamous cell carcinomas (ESCCs) have a superior prognosis than patients with HPV-negative ones, with a 63% reduction in overall mortality. However, the biologic basis for the improved survival among the HPV-positive patients is unclear and warrants further study.

Though not statistically significant, HPV infection was associated with improved survival in prospective studies, but was associated with poor survival in retrospective studies. The same phenomenon was also seen in studies containing large samples and relatively small samples. Future large prospective studies should be performed to confirm the association between HPV infection and esophageal cancer prognosis. When stratified by geographic region, HPV infection was not significantly associated with improved survival in China. However, most studies are carried out in Taiwan, so we should be cautious with the representativeness of these included studies.

Over 100 HPV types are classified as low-risk and high-risk based on their ability to induce malignant transformation of epithelial cells.^[35]^ The overall HPV prevalence in ESCC is 22.2%, wherein HPV-16 is the main high-risk genotype with a prevalence of 11.4%.^[11]^ Results from subgroup analyses stratified by HPV type showed that high-risk HPV or HPV-16 infection was associated with improved survival, whereas low-risk HPV infection was associated with poor survival. Due to the degree of heterogeneity in HPV genotyping amongst the 10 studies included in this meta-analysis, it was not possible to compare survival differences by HPV genotype. More studies are needed to analyze the survival difference between different HPV genotypes in ESCC.

Smoking is associated with a poor outcome of ESCC.^[36]^ Additional variables of potential prognostic importance include weight loss, anemia, performance status, dietary habits, and sexual behavior. However, only 2 studies reported the adjusted HRs, which showed that HPV infection was associated with improved survival. Other HRs were estimated from survival curves, which showed that HPV infection was associated with poor survival. So, future studies should be encouraged to adjust for other potential prognostic factors when comparing survival outcomes.

The present study has several strengths. First, the present meta-analysis is the first to examine OS differences in HPV-positive and HPV-negative esophageal cancers. Second, we applied a rigorous inclusion/exclusion criteria and advanced meta-analysis of HR for survival. Moreover, subgroup analyses stratified by the study type, geographical region, number of patients, pathological type, detection method, HPV type, maximum follow-up time, case diagnosis method, and the source of HR were conducted. Therefore, the effect of potential confounders was minimized. Furthermore, no publication bias was observed in this meta-analysis, combined with the results of the sensitivity analysis, indicating that our results are robust.

However, we recognize several limitations of the present meta-analysis. First, the estimates of HPV infection might be influenced largely by the sensitivity and accuracy of HPV DNA detection method and HPV types covered by the method. Therefore, to some extent, potential bias could not be completely excluded considering that different methods have been used in the included studies. Second, significant heterogeneity was observed. So, we used the meta-analysis with random-effects model to combine data whenever significant heterogeneity was found. Besides, appropriate well-motivated inclusion criteria were used to maximize homogeneity, and sensitivity and subgroup analyses were performed to investigate potential sources of heterogeneity. Third, the included studies were restricted to those published in English in our study, which might introduce language bias as well. Finally, only 20% included studies reported the adjusted HRs, which might cause residual confounding by other potential prognostic factors such as smoking.

## 5 Conclusions

In conclusion, the findings of this systematic review and meta-analysis indicated that HPV infection was not associated with improved survival in esophageal cancer patients. However, HPV-16-positive patients might have a significantly favorable survival. Considering the limitations of the present meta-analysis, further large prospective studies are encouraged to stratify survival analysis by HPV type.

## Supplementary Material

Supplemental Digital Content
