# Supplementary material for: Human papillomavirus-related esophageal cancer survival: A systematic review and meta-analysis
Source: Medicine (Baltimore). 2016 Nov 18;95(46):e5318. doi: 10.1097/MD.0000000000005318 (PMC5120915; doi:10.1097/MD.0000000000005318)
Supplement: Supplemental Digital Content [file medi-95-e5318-s001.docx]

**SUPPLEMENTARY 1. Search terms used in the meta-analysis**

Esophageal Neoplasms [MeSH Terms] AND (HPV [Title/Abstract] OR human papillomavirus [Title/Abstract]) AND (survival [Title/Abstract] OR prognosis [Title/Abstract] OR prognostic [Title/Abstract])

Limits: Publications in English
